# Role of Conductivity in the Ecotoxicological Effects of Lyophilized Graphene Oxides on *Lactuca sativa* and *Allium cepa*

**DOI:** 10.1021/acsomega.5c13552

**Published:** 2026-04-27

**Authors:** Wilfredo Rondan Huaman, María Rivera, Ulises Reno, Josefina Schmuck, Luciana Regaldo, Kauã Habache Farias, Jaime Zamora, Ana Maria Gagneten, Ana Champi

**Affiliations:** a Laboratorio de Novos Materiais de Carbono: Grafeno, 74362 Universidad Federal do ABC, 09210-580 Santo André, SP, Brazil; b Laboratorio de Ecotoxicología, Facultad de Humanidades y Ciencias, 28247 Universidad Nacional del Litoral, Santa Fe 3000, Argentina; c Consejo Nacional de Investigaciones Científicas y Tecnológicas (CONICET), CCT Santa Fe, Santa Fe S3000ADQ, Argentina

## Abstract

The ecotoxicity of graphene oxide (GO) remains insufficiently characterized, particularly regarding how synthesis parameters influence its environmental behavior. In this study, we investigated the physicochemical properties and biological effects of GO synthesized by a modified Hummer’s method and exposed to two oxidative interaction times with hydrogen peroxide (H_2_O_2_) for 1 h (1h-LSGO) and 5 h (5h-LSGO) under identical sonication conditions. GO was characterized by UV–vis spectroscopy, FTIR-ATR, zeta potential, electrical conductivity, and atomic force microscopy (AFM). Ecotoxicological responses of 1h-LSGO and 5h-LSGO were evaluated in *Lactuca sativa* and *Allium cepa*, focusing on germination, root elongation, antioxidant activity, chromosomal aberrations, and mitotic index, mainly due to the effects of GO conductivity. Treatment with a shorter oxidative interaction (1h-LSGO) produced GO with higher oxygen content (21.2%), partial restoration of sp^2^ domains (7.6%), conductivity (0.0249 mS·cm^–1^), and a more negative zeta potential (−40.1 mV). Meanwhile, the longest interaction (5h-LSGO) favored partial restoration of sp^2^ domains (48.1%), showing ∼20-fold higher conductivity (0.485 mS·cm^–1^) and zeta potential (−32.2 mV). Both materials caused only slight effects on seed germination, but their sublethal responses differed between species: 1h-LSGO significantly inhibited *L. sativa* root elongation (30%) and modulated antioxidant activity, while 5h-LSGO reduced *A. cepa* root growth (60%), decreased the mitotic index, and increased chromosomal aberrations at 100 mg L^–1^ (*p* < 0.05). In summary, the oxidative interaction time of H_2_O_2_ was revealed as a critical parameter that controls the surface chemistry of GO, its electronic structure, its conductivity, and its ecotoxic potential, with higher conductivity in 5h-LSGO correlating with stronger genotoxicity in *A. cepa* (e.g., reduced mitotic index and increased chromosomal aberrations at 100 mg L^–1^, *p* < 0.05).

## Graphical abstract

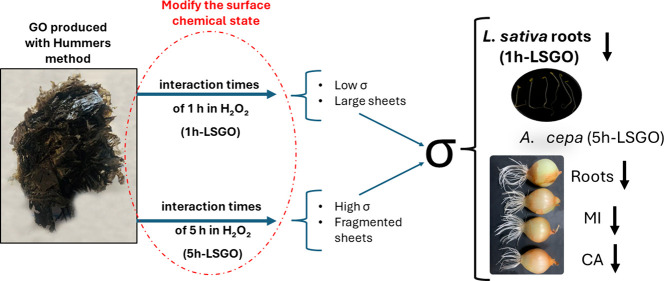

## Highlights

Holistic ecotoxicity assessment based on physicochemical characterization
GO conductivity from H_2_O_2_ exposure drives key ecotoxicity effects
Species-specific detoxification strategies in plants
Standardized synthesis protocols needed for safer environmental applications

## 1 Introduction

Graphene oxide (GO), a two-dimensional carbon-based nanomaterial, has attracted considerable attention due to its large specific surface area, tunable surface chemistry, and outstanding mechanical, electrical, and thermal properties, enabling applications in electronics, biomedicine, and environmental remediation.^1,2^ Among the synthesis parameters known to influence GO behavior, the oxidative interaction time with hydrogen peroxide (H_2_O_2_) has emerged as a critical factor because it modulates oxygen functional groups, colloidal stability, and reactivity toward biological interfaces.^3,4^ Changes in the oxidative treatment are expected to modulate the relative abundance of hydroxyl, epoxy, carbonyl, and carboxyl groups on the GO surface, thereby altering its surface charge, aggregation state, and interaction with plant cell walls.^5^

Plant bioassays using *L. sativa* and *A. cepa* are widely recognized as sensitive and cost-effective tools in standardized ecotoxicity testing.^6,7^ These species provide robust end points such as germination, root elongation, chromosomal aberrations, and mitotic index, allowing the integrated evaluation of phytotoxic and genotoxic effects of chemicals and nanomaterials.^1,8,9^ Nevertheless, few studies have systematically related defined synthesis parameters of GO, such as H_2_O_2_ oxidative interaction time, to its phytotoxicity and genotoxicity in multiple plant models, limiting the optimization of safer synthesis protocols and the interpretation of ecotoxicological data.^6,10^

In particular, most available studies employ GO produced under a single set of conditions without disentangling how controlled changes in oxidative treatment alter surface chemistry and, consequently, plant responses. Experimental evidence indicates that parameters such as oxidation degree, defect density, and surface charge affect GO aggregation, cell wall interactions, and the generation of reactive oxygen species in plants.^11^ However, the specific contribution of the H_2_O_2_ interaction time, while keeping sonication and other processing steps constant, remains poorly understood.

According to our knowledge, no previous study has systematically compared GO samples produced with different oxidative interaction times with H_2_O_2_, maintaining identical sonication and postsynthesis conditions, while simultaneously evaluating their phytotoxic and genotoxic effects on *L. Sativa* and *A. cepa*. This work integrates a detailed physicochemical analysis with additional parameters beyond standard germination and root elongation tests, including antioxidant activity, chromosomal aberrations, and mitotic index, thus offering a more mechanical and comprehensive perspective on how a synthesis parameter influences plant responses to GO.

In this study, we synthesized GO via a modified Hummer’s method and subsequently exposed the material to two different H_2_O_2_ oxidative interaction times (1 and 5 h) under identical sonication conditions, generating the samples of 1h-LSGO and 5h-LSGO, for later use in the experiments, at concentrations in the range 1.0 to100 mg L^–1^. The materials were characterized by UV–vis spectroscopy, FTIR-ATR, zeta potential, electrical conductivity, and atomic force microscopy to establish their structural and surface properties. Phytotoxic effects were subsequently assessed in *L. sativa* by examining germination, radicle elongation, fresh weight, and antioxidant activity. Ecotoxicological responses were further evaluated in *A. cepa* through measurements of root length, fresh weight, and antioxidant activity as well as genotoxicity determined by chromosomal aberrations and mitotic index. Our objectives were to relate differences in oxidative interaction time to changes in the physicochemical properties of GO and to correlate these changes with species-specific toxic outcomes, thus providing a mechanistic basis for understanding how synthesis conditions govern the ecotoxic potential of GO.

## 2 Methods

### 2.1 Characterization of GO

GO (1h-LSGO and 5h-LSGO) was synthesized from graphite powder according to a modified Hummer’s method and our previous works.^4,14^ The obtained GO dispersion was divided into two aliquots and subjected to different oxidative interaction times with hydrogen peroxide (H_2_O_2_; 1 and 5 h) to obtain 1h-LSGO and 5h-LSGO, respectively. All subsequent washing, neutralization, and sonication steps were performed under identical conditions for both samples, ensuring that the H_2_O_2_ oxidative interaction time was the only synthesis parameter intentionally varied. Absorption spectra of GO (1h-LSGO and 5h-LSGO) suspended in water were obtained using an Evolution One Plus UV–Vis spectrometer in the wavelength range of 200–800 nm. The zeta potential of the GO suspensions was measured using a Zetasizer NanoZS (Malvern Instruments Ltd., Malvern, UK). Physicochemical analysis was performed using FTIR-ATR (Fourier Transform Infrared Spectrometer 640-IR), and surface morphology was evaluated by atomic force microscopy (AFM/SPM Series 5500). For scanning electron microscopy (SEM) analysis, the FEI Quanta 250 environmental scanning electron microscope (ESEM) equipment is used.

### 2.2 Plant Material and Growth Conditions

#### 2.2.1 Germination Percentage and Radicle Elongation of *L. sativa*

Seeds of *L. sativa* were obtained from ISLA (variety ″Alface Emba Crespa”, Brazil). For germination, a chamber was built by our team that controls temperature (fixed at ∼25 °C) and humidity (60%). The germination assay was conducted following standardized protocols from USEPA guidelines to evaluate the acute phytotoxicity of 1h-LSGO and 5h-LSGO on *L. sativa*.^12^ The pH of the control and GO test solutions was adjusted to a physiological range before plant exposure (6.0 ± 0.2), which is considered optimal for reliable and standardized seed germination tests,^13^ at the beginning of the experiment, as recommended by USEPA (1989).^12^ These seeds were surface sterilized with 1% sodium hypochlorite for 5 min, rinsed thoroughly with deionized water, and evenly distributed in Petri dishes (20 seeds per dish) lined with filter paper. Test solutions were prepared by dispersing GO (1h-LSGO or 5h-LSGO) in deionized water at concentrations of 0.1, 1, 10, and 100 mg L^–1^, with a negative control (deionized water only). Each treatment and control were replicated three times (*n* = 3).

The experiment was conducted under static conditions in a controlled environment chamber at 22 ± 1 °C and 60% relative humidity in a dark chamber for 7 days. Germination was monitored daily, with seeds considered germinated upon radicle emergence ≥2 mm. Germination percentage (G%) was calculated using eq 1:

$$\mathrm{germination}\left(\%\right)=\frac{\left(\mathrm{number}\;\mathrm{of}\;\mathrm{germianted}\;\mathrm{seeds}\right)}{\mathrm{total}\;\mathrm{seeds}}\times 100$$ 1

The radicle length of *L. sativa* was measured after 120 h of exposure by using a digital caliper, and the mean radicle length (mm) was calculated for each Petri dish. Negative control (deionized water) was included in all experiments and developed under the same experimental conditions.

#### 2.2.2 Fresh Weight Measurements of *L. sativa*

Fresh weight measurements were conducted for both *L. sativa* seedlings and *A. cepa* bulbs to evaluate biomass changes under different GO concentration exposures. For *L. sativa*, seedlings were carefully removed from Petri dishes, gently rinsed with deionized water to remove residual GO, blotted dry with filter paper, and immediately weighed (mg) using an analytical balance (precision ± 0.1 mg).

#### 2.2.3 Pre-Germination of *A. cepa*

For the *A. cepa* experiment, bulbs with similar characteristics (weight ∼ 90 g and shape) were used and placed suspended in 150 mL beakers in a dark environment and with air pumps to maintain oxygenation in the beakers. During the pregermination process of *A. cepa* bulbs, the following experimental design was used: (3 bulbs × 3 dilutions × 2 types of GO) + 3 negative-control bulbs, totaling 21 bulbs. Bulbs were cleaned and washed without damaging the primordial roots and then placed in beakers with mineral water, covering only the basal plate to the height of the roots to allow the development of the roots, in darkness at room temperature. After 72 h, when bulbs had roots between 2 and 3 cm, they were exposed to different concentrations (1–100 mg L^–1^) of GO (1h-LSGO and 5h-LSGO) and to a negative control, for genotoxicity and cytotoxicity analyses.^14^

#### 2.2.4 Root Length and Fresh Weight of *A. cepa*

*A. cepa* bulbs were weighed before and after the 96 h exposure period using the same protocol, with root systems gently separated from bulbs and weighed independently to assess tissue-specific effects. All measurements were performed in triplicate (*n* = 3) under controlled laboratory conditions (20.8 ± 0.7 °C, 62 ± 9% humidity) to minimize environmental variability. The root length relative to control (RLRC) was calculated to compare the effects of GO treatments to negative control, following standardized phytotoxicity evaluation protocols.^15,16^ For each treatment, the mean root length (cm) was measured after the exposure period and normalized to the corresponding mean root length of the negative control according to the following equation:

$$\mathrm{RLRC}=\frac{\mathrm{mean}\;\mathrm{root}\;\mathrm{length}\;\mathrm{in}\;\mathrm{treatment}}{\mathrm{mean}\;\mathrm{root}\;\mathrm{length}\;\mathrm{in}\;\mathrm{control}}\times 100$$ 2

where values below 100% indicate growth inhibition, and values above 100% indicate stimulation relative to control conditions.

### 2.3 Antioxidant Activity of *L. sativa* and *A. cepa*

For evaluating the effect of GO (1h-LSGO and 5h-LSGO) on the antioxidant potentials of *L. sativa* and *A. cepa*, the 2,2-diphenyl-1-picrylhydrazyl radical (DPPH) assay was carried out.^17–19^ In this investigation, 50 μL of each methanolic extract of *L. sativa* and *A. cepa* treated with GO at concentrations of 0.1, 1, 10, and 100 mg L^–1^ was mixed with DPPH radical solution in methanol (0.1 mM, 150 μL). All samples were covered with aluminum foil and incubated at room temperature in the dark for 30 min. The absorbance at 517 nm was then recorded by using an Evolution One Plus UV–Vis spectrophotometer. DPPH reagent in methanol without leaf extract was used as a control, and the percentage radical scavenging activity (DPPH %) was determined as^20^

$$\mathrm{DPPH}\%=\left(\frac{A_{0}-A_{1}}{A_{0}}\right)\times 100$$ 3

where *A*_0_ is the absorbance of blank samples without added extract (nonsample DPPH solution) and *A*_1_ represents the absorbance in the presence of extract at 517 nm.

### 2.4 Mitotic Index and Genotoxicity of *A. cepa*

Roots (2–3 cm) of *A. cepa* were treated with 1h-LSGO and 5h-LSGO suspensions at 1, 10, and 100 mg L^–1^, as well as using distilled water as a control. For each treatment, roots from three bulbs were collected after 24 h exposure, fixed in Carnoy’s solution (absolute ethanol:glacial acetic acid, 3:1) for 24 h at 4 °C, washed, and stored in 70% ethanol. Roots were hydrolyzed in 1 N HCl (60 °C, 8–10 min) and stained with 2% aceto-orcein, and the meristematic region of each root was cut into 1–2 mm segments and squashed in 45% acetic acid. Slides were coded randomly, observed under a light microscope (100×), and ∼9000 cells per treatment were analyzed to evaluate cytotoxicity, genotoxicity, and mutagenicity. MI was used as an indicator of cytotoxicity associated with the inhibition of cell division. The MI was calculated as the ratio between the number of dividing cells and the total number of cells, multiplied by 100:

MI=dividingcells/totalcells×100 4

Chromosome alterations were determined in different cell cycle phases (prophase, metaphase, anaphase, and telophase) by evaluating several types of aberration (e.g., chromosomal breaks, bridges, losses, and delays) and used to determine genotoxicity.^2,15^

### 2.5 Data Analysis

The biological data obtained from the *L. sativa* and *A. cepa* trials were analyzed by one-way analysis of variance (ANOVA) after verification of homoscedasticity. To identify significant differences between controls and the evaluated concentrations, Dunnett’s post hoc test was applied, whereas Tukey’s test was used for multiple comparisons between groups, considering *p* < 0.05 as the significance threshold. The analyses were performed with SigmaPlot 12.0 software, and graphs were generated using OriginPro 2021.

Data normality was assessed using the Shapiro–Wilk test (all data sets passed, *p* > 0.05). The *p*-value threshold of 0.05 was selected without multiple comparison correction, as the study focuses on exploratory pairwise comparisons via Tukey’s tests.

## 3 Results and Discussion

### 3.1 Characterization of GO

The UV–vis absorption spectra of 1h-LSGO and 5h-LSGO revealed distinct structural differences influenced by the interaction time of H_2_O_2_ (Figure 1).

**Figure 1:**
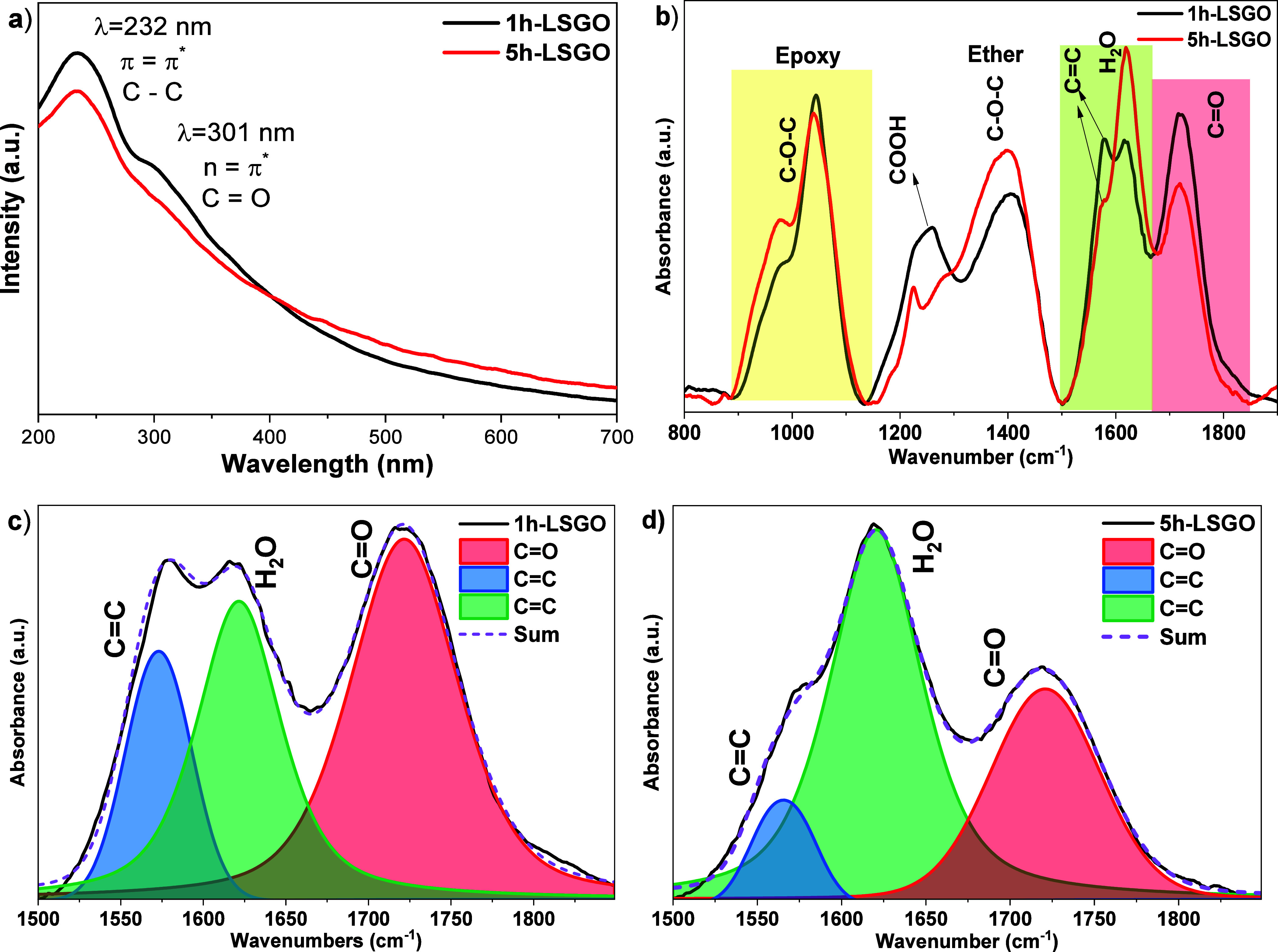
UV–vis spectra of (a) 1h-LSGO and 5h-LSGO and (b) FTIR-ATR of 1h-LSGO and 5h-LSGO. (c, d) Normalized FTIR plot.

For 1h-LSGO, a prominent absorption peak at 230 nm was observed, corresponding to π–π* transitions of sp^2^ conjugated carbon domains, characteristic of partially reduced graphene oxide (rGO).^1^ In contrast, 5h-LSGO showed a small shoulder shifted toward the red at around 300 nm, indicative of a reduction in the *n*–π* transitions of the oxygenated functional groups, suggesting that prolonged interaction with H_2_O_2_, together with sonication, increased oxidative cleavage and introduced more defects.^1,5^ Notably, 1h-LSGO demonstrated higher absorbance intensity at 230 nm compared to 5h-LSGO, which, together with the more pronounced shoulder near 300 nm, is consistent with a higher density of oxygenated moieties in 1h-LSGO and a somewhat more graphitic character in 5h-LSGO. These findings align with zeta potential data (Table 1), where 1h-LSGO exhibited greater colloidal stability (−40.1 mV at 0.1 mg L^–1^) than 5h-LSGO (−32.2 mV), likely due to its retained aromatic structure and reduced aggregation propensity in aqueous media.

**Table 1:** FTIR Data and Oxygen-Related Bonds for 1h-LSGO and 5h-LSGO

	position (cm)	area	(C=C + H_2_O)/C=O	ORB
1h-LSGO	1573.0	0.064	1.01	76.7%
	1621.6	0.132		
	1721.7	0.194		
5h-LSGO	1565.6	0.022	2.00	73.6%
	1620.6	0.212		
	1720.8	0.117		

Figure 1b and Figure S1c show the FTIR-ATR spectra of the 1h-LSGO and 5h-LSGO samples, where the main vibrational contributions were identified at ∼1050 cm^–1^ (C–O–C, epoxy), ∼1250 cm^–1^ (COOH), ∼1400 cm^–1^ (C–O–C, ether), 1550 cm^–1^ (C=C), 1620 cm^–1^ (H_2_O), and 1720 cm^–1^ (C=O).^21^ These positions are consistent with the expected vibrational modes of the graphitic domains and oxygenated functional groups. On the other hand, deconvolution of the FTIR absorbance spectra (Figure 1c,d) was performed using a pseudo-Voigt function in order to resolve the overlapping contributions of the C–C, C=C, and C=O vibrational modes (Table S1, Supporting Information). From these deconvoluted peaks, the relative areas were quantified and are summarized in Table 1. The (C=C + H_2_O)/C=O ratio was calculated as an indicator of the oxidation degree of the samples. A lower ratio reflects a higher contribution of carbonyl groups, consistent with a more oxidized structure, whereas a higher ratio indicates a predominance of nonoxygenated carbon bonds.

In addition, the contribution of oxygen-related bands (ORB) was estimated according to the following expression:^22^

$$\mathrm{ORB}=\frac{\left(\sum {\mathrm{area}}_{800-1850}-{\mathrm{area}}_{C=C+H_{2}O_{2}}\right)}{\sum {\mathrm{area}}_{800-1850}}\times 100$$ 5

According to the (C=C + H_2_O)/C=O relationship, values of 1.01 and 2 were obtained for 1h-LSGO and 5h-LSGO, showing that prolonged treatment with H_2_O_2_ reduced functional groups that contain oxygen, corroborated in a previous work with the XPS technique.^4^ These results are further confirmed using the formula for obtaining the ORB, yielding values of 76.7 and 73.6% for 1h-LSGO and 5h-LSGO, respectively, and are within the range of values obtained by other authors.

Table 2 presents the zeta potentials of 1h-LSGO and 5h-LSGO dispersions in water at varying concentrations ranging from 0.1 to 100 mg L^–1^. Both materials exhibit negative zeta potential values across all tested concentrations, reflecting the presence of negatively charged surfaces arising from ionized oxygen containing functional groups, such as carboxyl and hydroxyl moieties.^23^ 1h-LSGO, characterized by a higher proportion of sp^3^ hybridized carbon and consequently greater oxygenation, displays more negative zeta potentials compared to 5h-LSGO (∼10% sp^3^), as reported in our previous work.^24^ Specifically, zeta potentials for 1h-LSGO range from −40.1 ± 2.2 mV at 0.1 mg L^–1^ to −22.5 ± 3.1 mV at 100 mg L^–1^, while those for 5h-LSGO span −32.2 ± 3.1 to −15.4 ± 1.8 mV. This enhanced negativity in 1h-LSGO suggests superior colloidal stability in aqueous media, as absolute zeta potential values exceeding ∼30 mV are generally indicative of kinetic stability against aggregation.^25–28^

**Table 2:** Zeta Potential of 1h-LSGO and 5h-LSGO for the Different Concentrations of 1h-LSGO and 5h-LSGO^a^

		zeta potential (mV)			
sample	water	0.1 mg L^–1^	1 mg L^–1^	10 mg L^–1^	100 mg L^–1^
1h-LSGO	0 ± 1.7	–40.1 ± 2.2	–31.2 ± 1.6	–27.1 ± 1.5	–22.5 ± 3.1
5h-LSGO	0.1 ± 3.1	–32.2 ± 3.1	–29.4 ± 1.8	–26.3 ± 1.9	–15.4 ± 1.8

a The figures express the media and the standard deviation.

In Table 3, conductivity values increase with concentration for both 1h-LSGO and 5h-LSGO, consistent with greater particle density facilitating charge transport. However, 5h-LSGO demonstrates markedly superior conductivity, with values rising from 0.112 mS·cm^–1^ at 0.1 mg L^–1^ to 0.485 mS·cm^–1^ at 100 mg L^–1^, compared to 0.0064 mS·cm^–1^ to 0.0249 mS·cm^–1^ for 1h-LSGO. This disparity is primarily due to the higher sp^2^ content (∼48%) in 5h-LSGO.^24^ In contrast, the elevated sp^3^ hybridization in 1h-LSGO disrupts these conductive pathways, resulting in semi-insulating behavior akin to highly oxidized graphene forms.^29^

**Table 3:** Conductivity of 1h-LSGO and 5h-LSGO for Different Concentrations of 1h-LSGO and 5h-LSGO Compared with Water^a^

	conductivity (mS·cm^–1^)				
sample	water	0.1 mg L^–1^	1 mg L^–1^	10 mg L^–1^	100 mg L^–1^
1h-LSGO	0.0020	0.0064	0.0091	0.0172	0.0249
5h-LSGO	0.0019	0.112	0.174	0.291	0.485

a All measurements were performed at room temperature.

The concentrations of thiol (−SH) functional groups were determined by UV–vis spectroscopy for both types of GO. The results show that the material treated for 1 h (1h-LSGO) had a thiol concentration of 7.2 μM, while the material treated for 5 h (5h-LSGO) reached a slightly higher concentration of 7.7 μM. This variation suggests that the duration of interaction with H_2_O_2_ during the synthesis of the GO influences the density of thiol groups present on the GO surface and could have implications for its chemical reactivity and potential ecotoxicological behavior.^30,31^

Figure 2 and Figure S1a,b show atomic force microscopy (AFM) images and height profiles of two GO variants synthesized with different H_2_O_2_ interaction times: 1h-LSGO (1 h) and 5h-LSGO (5 h). 1h-LSGO exhibits large, flat sheets with lateral dimensions of several micrometers and a uniform thickness of ∼12–16 nm, indicative of thin stacks of few-layer GO sheets. In contrast, 5h-LSGO exhibits significantly smaller (0.5–2 μm) (Figure S2, Supporting Information), fragmented sheets with irregular edges, greater roughness, and the presence of folds and local aggregates, reflecting more aggressive oxidation that disrupts the carbon plane and introduces structural defects. These morphological differences confirm that the interaction time with H_2_O_2_ is a critical parameter in GO synthesis, directly modulating its size, homogeneity, and colloidal stability. 1h-LSGO, with its larger intact surface area, promotes more effective interactions with cell membranes, explaining its greater inhibition of root elongation in *L. sativa*. Meanwhile, fragmentation of 5h-LSGO increases its surface reactivity and penetration capacity into meristematic tissues, correlating with more pronounced cytogenotoxic effects in *A. cepa*, especially at 100 mg L^–1^.

**Figure 2:**
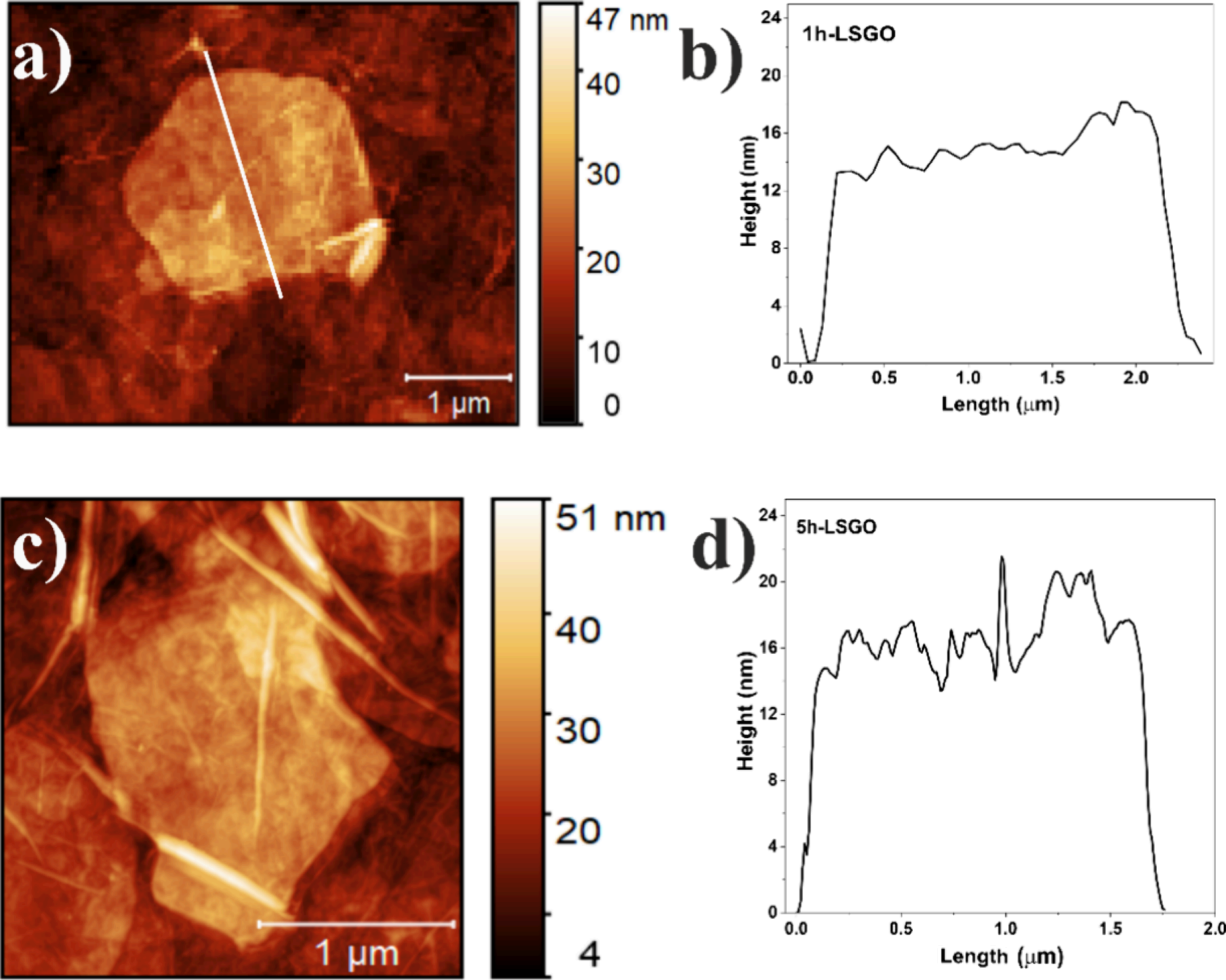
AFM images with corresponding height profiles (right) of GO: (a) 1h-LSGO, (b) height profiles 1h-LSGO, (c) 5h-LSGO, and (d) height profiles 5h-LSGO.

Following, SEM was employed to quantify the sheet size distribution of the 5h-LSGO (Figure S2, Supporting Information), displaying a normal (Gaussian) distribution with a mean size of 0.535 ± 0.192 μm, indicating a relatively homogeneous population of smaller sheets. This underscores the impact of prolonged H_2_O_2_ exposure on fragmentation, which likely enhances the material’s dispersibility and biological interactions.

In summary, the multitechnique characterization shows that 1h-LSGO is a more oxidized, highly charged, and colloidally stable material with low conductivity and larger, continuous sheets, whereas 5h-LSGO exhibits slightly lower oxygen content, higher conductivity, increased thiol-group density, and more fragmented morphology.

### 3.2 Effect of GO on Growth and Physiological Traits of *Lactuca sativa*

After exposing *L. sativa* seeds to 1h-LSGO (Figure 3), the final germination percentage remained similar to the control at all tested concentrations (0.1–100 mg L^–1^; *p* > 0.05), indicating that this end point is relatively insensitive to the presence of GO under the studied conditions. Likewise, no statistically significant differences in final germination were observed for seeds exposed to 5h-LSGO compared to the control, although slight fluctuations in germination percentages were recorded at the highest concentrations.

**Figure 3:**
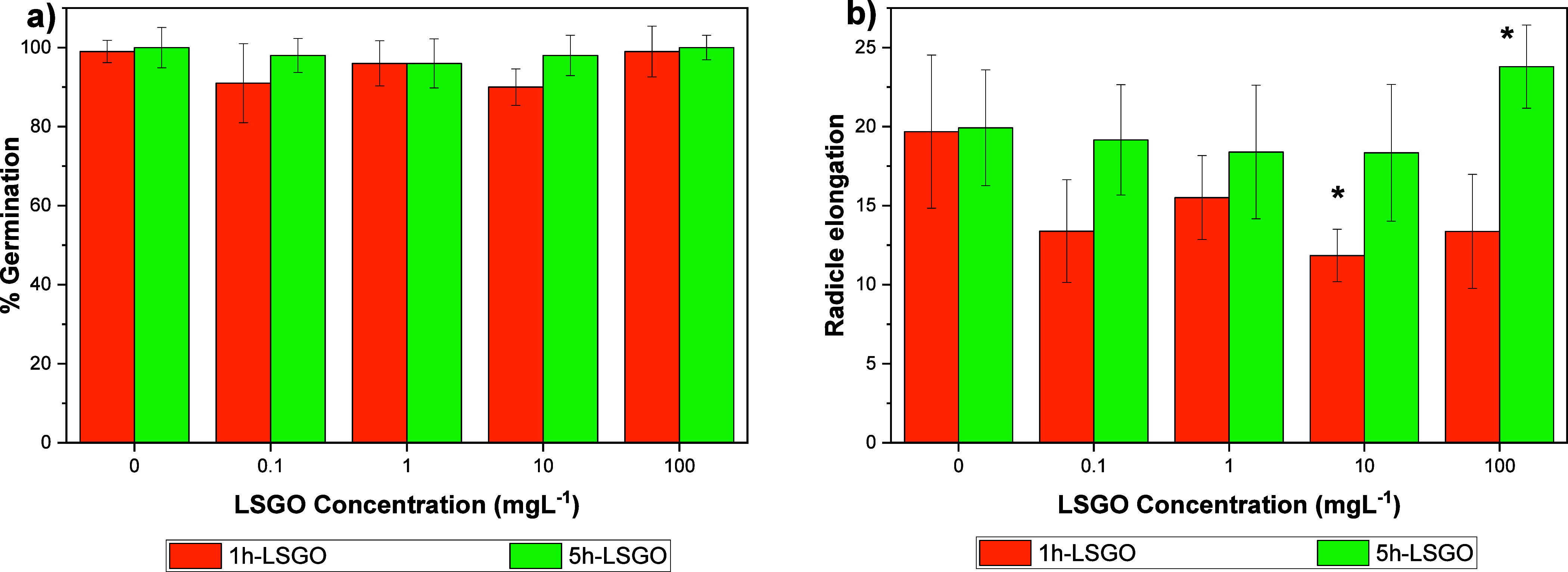
(a) Germination percentage and (b) radicle elongation of *L. sativa* exposed to different concentrations (0, 0.1, 1, 10, and 100 mg L^–1^) of 1h-LSGO and 5h-LSGO. Error bars indicate 1 standard deviation. (*) Statistically significant differences (*p* < 0.05).

Considering radicle elongation, *L. sativa* seedlings exposed to 1h-LSGO exhibited a significant reduction in root length at 100 mg L^–1^ (*p* < 0.05), while lower concentrations (0.1–10 mg L^–1^) did not differ from the control (Figure 3b). In contrast, no significant changes in radicle elongation were detected for any concentration of 5h-LSGO, suggesting that early root growth is more sensitive to the more oxidized GO sample than to the less oxidized, more conductive material.

These results indicate that differences in the time of oxidative interaction with H_2_O_2_, rather than sonication conditions, which were identical for both materials, have a central role in modulating the phytotoxicity of GO toward *L. sativa*. The more oxidized 1h-LSGO, characterized by a higher contribution of oxygen-related bonds (76.7%), more negative zeta potential (∼−40.1 mV), and lower electrical conductivity (0.0249 mS·cm^–1^), is expected to display stronger electrostatic interactions and adhesion to the root surface, favoring local accumulation and interference with water and nutrient uptake.^2^ In contrast, 5h-LSGO, which has slightly less oxygen-related bonds (73.6%), is more conductive (0.485 mS·cm^–1^) and morphologically more fragmented and appears to interact less aggressively with *L. sativa* radicles at the concentrations tested. This pattern is consistent with previous studies showing that highly oxidized and strongly charged GO can induce root growth inhibition and oxidative stress in plants.^3^

On the other hand, 5h-LSGO, slightly less dispersed (zeta potentials below −30 mV for 1, 10, and 100 mg L^–1^), did not have a negative effect on different concentrations. This could be due to the agglomeration and fragmentation of GO sheets during prolonged exposure to H_2_O_2_ (Figure 2), which generates smaller (1.5 μm), defective structures with oxygen-related bonds and, consequently, reduced interaction capacity. These findings are consistent with previous studies in which GOs with oxygen content below 25% exhibited lower acute toxicity.^4^ As can be seen in Figures 3b and 4b, radicle elongation was positively favored with 5h-LSGO at a concentration of 100 mg L^–1^, supporting the hypothesis that greater exposure of GOs to H_2_O_2_ in the synthesis process favors interaction with this biological model.

**Figure 4:**
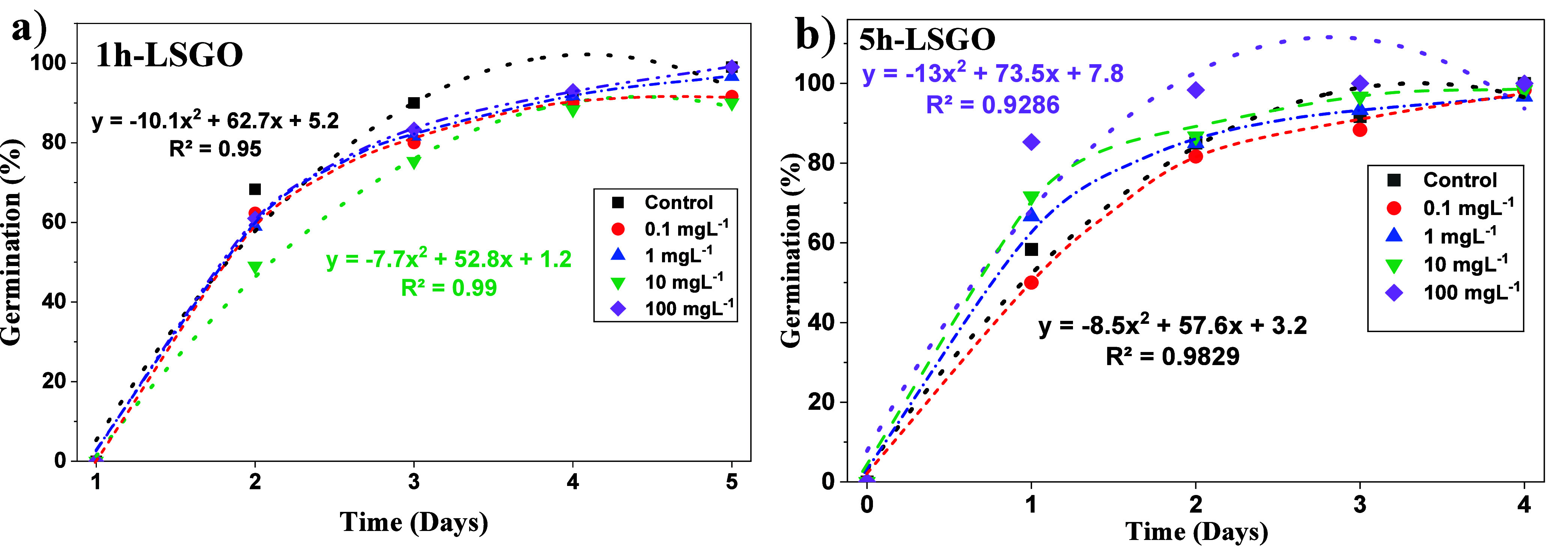
Germination percentage per day for *L. sativa* seeds exposed to different concentrations (0, 0.1, 1, 10, and 100 mg L^–1^) of (a) 1h-LSGO and (b) 5h-LSGO.

Figure 4 shows the daily germination percentages of *L. sativa* seeds with different concentrations of LSGO (0.1, 1, 10, and 100 mg L^–1^), modeling the data with quadratic functions (*y* = *ax* *^2^* + *bx* + *c*) to estimate the day of maximum germination using the vertex of the parabola: *x* = – *b*/2*a*. In 1h-LSGO (Figure 4a), the control reached its maximum on day ∼3.1 and the treatment with 100 mg L^–1^ on day ∼3.4, showing active but reduced germination, with the other concentrations having intermediate values. In 5h-LSGO (Figure 4b), the control reached its maximum on day ∼3.4, while the 100 mg L^–1^ treatment reached its maximum on day ∼2.0. This behavior suggests that 1h-LSGO may subtly alter the timing of germination without substantially affecting the final percentage, in agreement with the predominantly sublethal nature of its effects on root elongation.

### 3.3 Effect of GO on Growth and Physiological Traits of *Allium cepa*

After *A. cepa* bulbs were exposed to 1h-LSGO and 5h-LSGO, root fresh weight showed only slight variations at low and intermediate concentrations, whereas clear reductions were detected at the highest doses. For 1h-LSGO, the mean root fresh weight was 0.73 ± 0.23 g in the control and 0.99 ± 0.10, 0.80 ± 0.06, and 0.78 ± 0.17 g at 0.1, 1, and 10 mg L^–1^, respectively (*p* > 0.05) but dropped to 0.24 g at 100 mg L^–1^ (*p* < 0.05). For 5h-LSGO, root fresh weight was 0.76 ± 0.10 g in the control and 1.18 ± 0.31, 0.56 ± 0.09, and 0.32 ± 0.03 g at 0.1, 10, and 100 mg L^–1^, respectively, revealing a marked decrease of the root fresh weight at the highest concentrations. These patterns indicate that only the highest doses of both GOs notably affected biomass accumulation in the root system with a more pronounced reduction for 5h-LSGO.

Root length expressed as root length relative to the control (RLRC) is shown in Figure 5. After 5 days of exposure, RLRC values for both 1h-LSGO and 5h-LSGO were close to or slightly above 100% at the lowest concentration (1 mg L^–1^), indicating the absence of inhibitory effects and even slight stimulation of root growth at this level. However, at the highest concentration (100 mg L^–1^), a pronounced reduction in root growth was observed, with a significant inhibition (*p* < 0.001) that was especially evident for 5h-LSGO, where root length dropped to below 40% of the control. This pattern reveals a concentration-dependent phytotoxic effect of GO on *A. cepa* roots, more accentuated for the sample treated for 5 h with H_2_O_2_ (5h-LSGO). Similar effects were reported for flubendiamide (FLB) and pethoxamid herbicide on *A. cepa*.^2,15^

**Figure 5:**
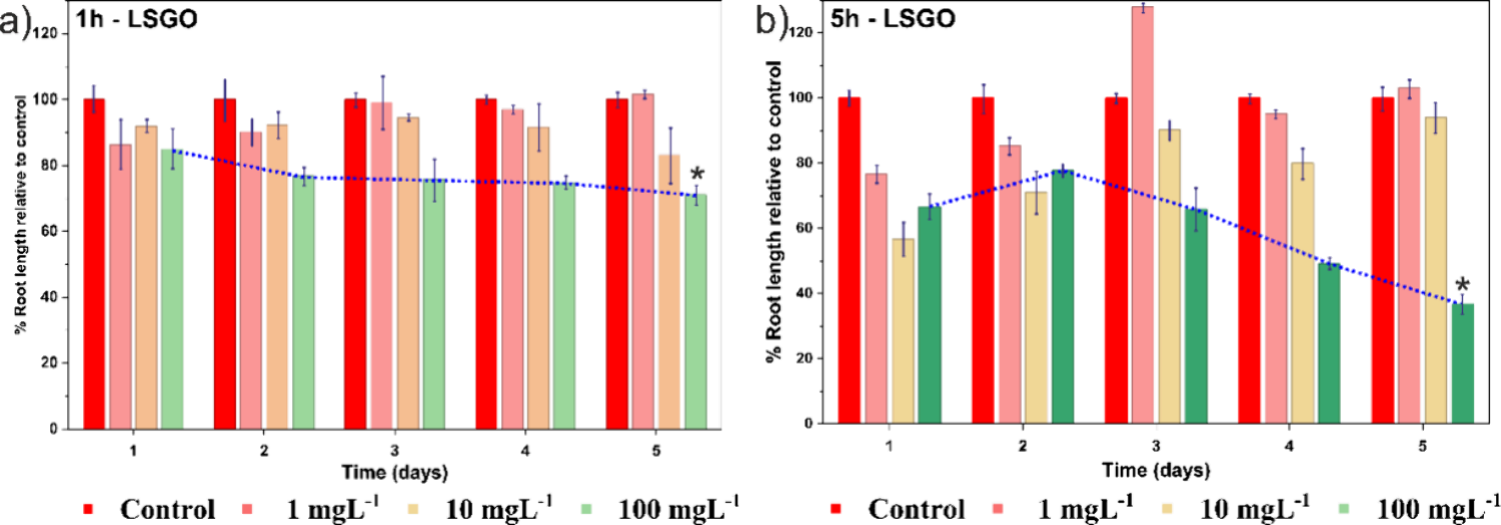
Root elongation of *A. cepa*. (a) Root length treated with 1h-LSGO relative to the control and (b) root length treated with 5h-LSGO relative to the control.

The greater inhibition induced by 5h-LSGO is consistent with its physicochemical profile. Increased fragmentation may favor closer contact of smaller GO sheets with the root surface and apoplastic spaces, while more extensive sp^2^ domains and higher conductivity could enhance electron transfer interactions at the interface, potentially contributing to a local redox imbalance.

The ∼20-fold higher conductivity of 5h-LSGO (0.485 mS·cm^–1^) compared with 1h-LSGO (0.0249 mS·cm^–1^) likely aggravates genotoxicity in *A. cepa* by promoting more efficient interfacial charge transfer, which can disturb cellular redox homeostasis and amplify oxidative stress. Importantly, 5h-LSGO is also markedly more conductive than rGO (0.0421 mS·cm^–1^), ∼12 × higher, underscoring that the 5 h material may enable stronger electron exchange at the bio-nano interface than conventional rGO.^32,33^ This behavior is consistent with the substantially higher sp^2^ fraction in 5h-LSGO (48.1%) relative to 1h-LSGO (7.6%), as a more graphitic network enhances electron delocalization and mobility, potentially perturbing membrane potential and mitochondrial electron-transport process effects previously reported for other highly conductive carbon-based nanomaterials. In contrast, the more oxidized, highly charged, and colloidally stable but less conductive 1h-LSGO may interact predominantly on the outer surface of the root, producing modest effects on elongation at 100 mg L^–1^ and an appreciable decrease in fresh weight without the severe growth suppression observed with 5h-LSGO.

Altogether, the growth and biomass data for *A. cepa* indicate that both GO samples can impair root development at concentrations (≥100 mg L^–1^) but that the less oxidized, more conductive, and morphologically more disrupted 5h-LSGO exerts a stronger inhibitory effect on root elongation than the more oxidized 1h-LSGO. These responses, which depend on the species and material, are consistent with the idea that subtle changes in the chemistry and morphology of the GO surface, driven in this case by the duration of oxidative interaction with H_2_O_2_, can substantially modify its phytotoxic potential in root apex tissues, effects that will be seen in the mitotic index section.

### 3.4 Antioxidant Activity in *L. sativa* and *Allium cepa* Assays

The DPPH radical scavenging assay revealed distinct antioxidant responses in *L. sativa* and *A. cepa* exposed to 1h-LSGO and 5h-LSGO (Figure 6). In *L. sativa* roots, exposure to 1h-LSGO caused only modest variations in DPPH scavenging at 0.1–10 mg L^–1^, with values remaining close to the control, whereas at 100 mg L^–1^, a tendency toward reduced radical scavenging was observed. This pattern suggests that, under the present experimental conditions, the antioxidant capacity of *L. sativa* is relatively resilient to 1h-LSGO at low and intermediate concentrations but may be partially compromised at the highest dose, consistent with the inhibition of radicle elongation observed at 100 mg L^–1^. In contrast, 5h-LSGO did not induce marked changes in DPPH values in *L. sativa* across the tested concentration range, indicating that the overall nonenzymatic antioxidant pool was not strongly affected by this GO variant.

**Figure 6:**
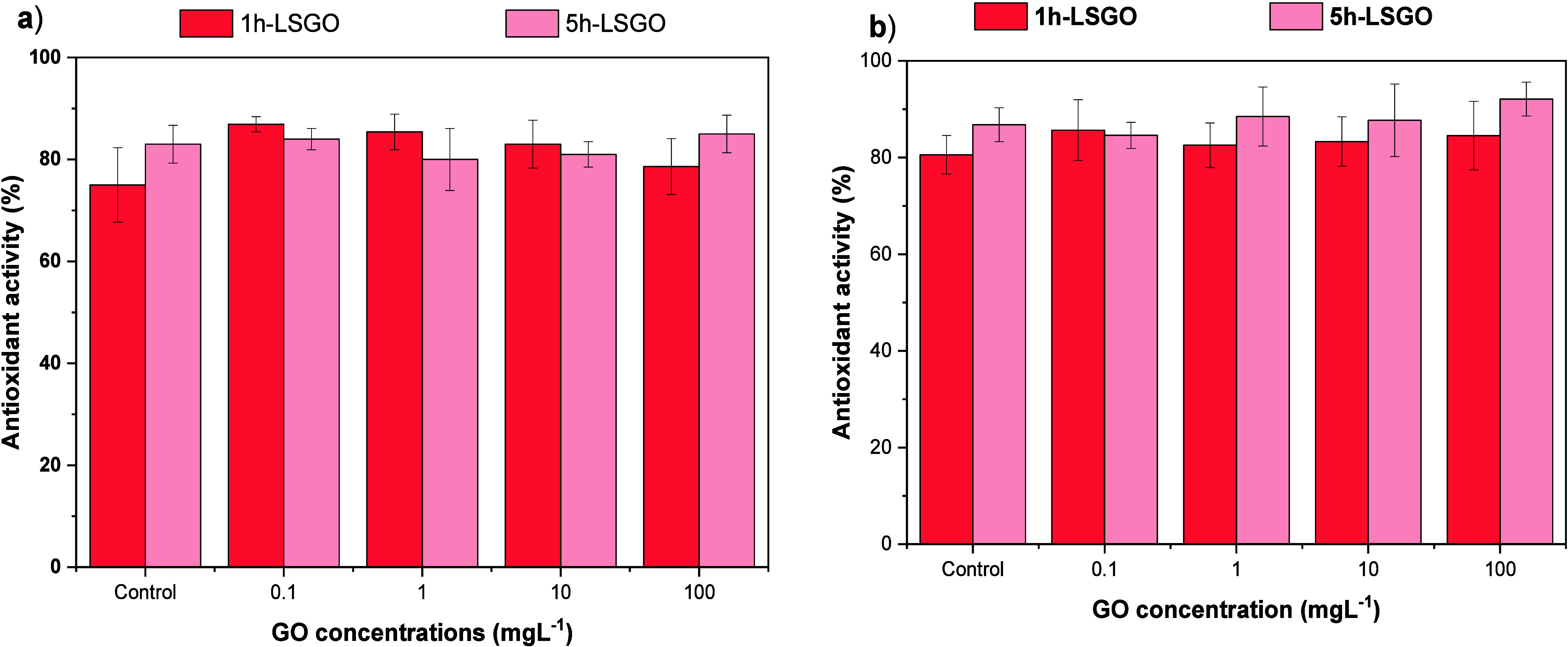
(a) Antioxidant activity of *L. sativa* roots exposed to different concentrations of GO (1h-LSGO and 5h-LSGO). (b) Antioxidant activity of *A. cepa* exposed to different concentrations of GO (1h-LSGO and 5h-LSGO). Error bars indicate 1 standard deviation.

In *A. cepa*, a different trend was detected. For 1h-LSGO, DPPH scavenging showed a concentration-dependent decrease, particularly at the highest tested concentration, indicating a reduction in the total radical scavenging capacity of root extracts. This decrease is in line with the growth and biomass data, suggesting that prolonged exposure to higher doses of 1h-LSGO can lead to consumption or impairment of antioxidant reserves in the root apex. In contrast, exposure to 5h-LSGO resulted in DPPH values that were similar to those of the control at low and intermediate concentrations with a slight increase at 100 mg L^–1^. Although this increase was modest, it may reflect an enhanced antioxidant response triggered by the stronger cytogenotoxic effects of 5h-LSGO at high concentration, as indicated by the more pronounced inhibition of root elongation and alterations in mitotic index.

Overall, the DPPH data support a scenario in which *L. sativa* shows limited modulation of nonenzymatic antioxidant capacity, with signs of depletion only under the highest 1h-LSGO concentration, whereas *A. cepa* exhibits a more pronounced, concentration-dependent response, particularly to 1h-LSGO and to high doses of 5h-LSGO. These species-specific patterns are consistent with the different sensitivities observed in growth and cytogenetic parameters and suggest that *A. cepa* activates antioxidant defense mechanisms similar to those described in cases of aluminum toxicity, in which the plant uses increased antioxidant enzyme activity and the accumulation of nanomaterials in root tissues to counteract stress.^34,35^

### 3.5 Mitotic Index of *Allium cepa*

Figure 7 presents the mitotic index distribution across the four mitotic phases (prophase, metaphase, anaphase, and telophase) in *A. cepa* root meristem cells treated with increasing concentrations (0.1, 1, 10, and 100 mg L^–1^) of 1h-LSGO and 5h-LSGO. Observed in Figure 7 and Table 4, the MI values reflected a typical proportion of actively healthy dividing cells in the control root meristems, with most mitotic figures concentrated in the prophase and metaphase.

**Figure 7:**
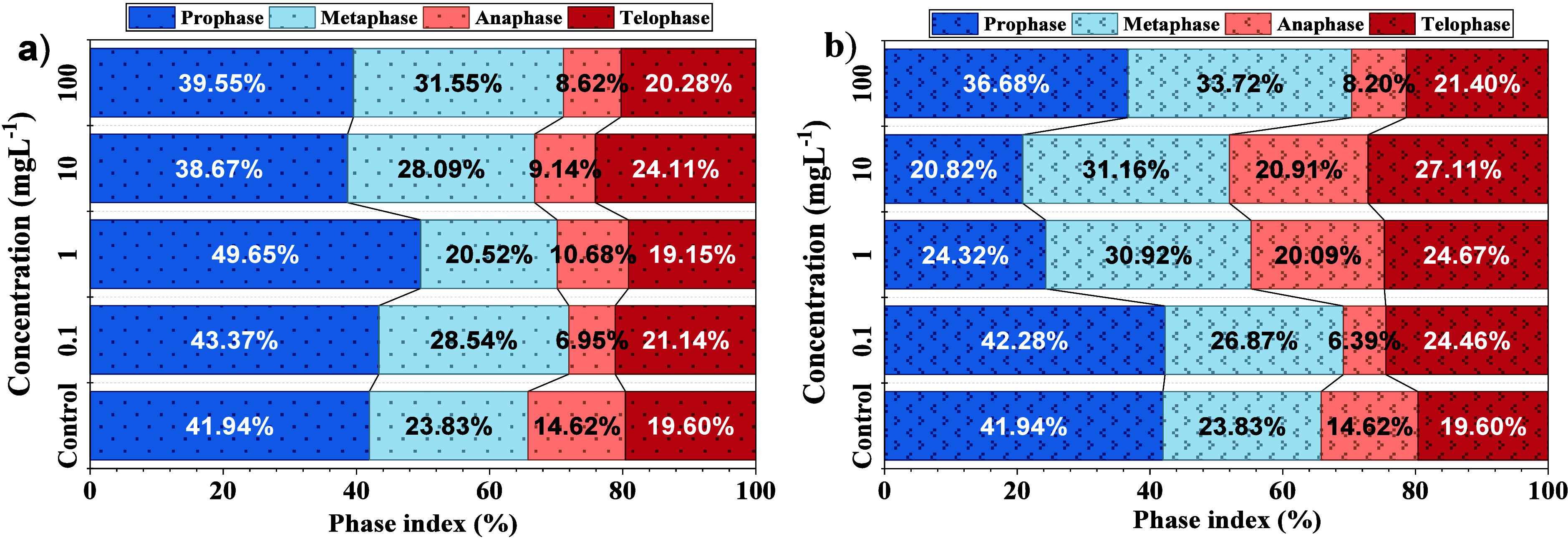
Phase index of *A. cepa* exposed to (a) 1h-LSGO and (b) 5h-LSGO.

**Table 4:** Effect of Exposure to Different Concentrations of 1h-LSGO and 5h-LSGO on Mitotic and Phase Index (%) in *A. cepa* Roots^a^

					phase index (%) ± SD^a^			
	concentration (mg L^–1^)	CCN	CA	MI ± SD	prophase	metaphase	anaphase	telophase
1h-LSGO	control	9035	3	4.23 ± 0.14	32.67 ± 5.96	26.37 ± 4.05	22.38 ± 2.54	18.58 ± 1.34
	0.1 mg L^–1^	9282	22	4.67 ± 0.75	43.37 ± 9.48	28.54 ± 5.73	6.95 ± 4.26	21.14 ± 4.03
	1 mg L^–1^	8968	14	6.32 ± 0.93	49.65 ± 10.04	20.52 ± 6.59	10.68 ± 3.51	19.15 ± 6.59
	10 mg^–1^	9384	18	4.49 ± 0.36	38.67 ± 12.85	28.09 ± 4.14	9.14 ± 5.84	24.11 ± 4.14
	100 mg^–1^	9521	54	4.47 ± 0.37	39.55 ± 7.09	31.55 ± 8.38	8.62 ± 2.23	20.28 ± 8.38
5h-LSGO	control	9035	3	4.23 ± 0.14	32.67 ± 5.96	26.37 ± 4.05	22.38 ± 2.54	18.58 ± 1.34
	0.1 mg L^–1^	9429	5	4.52 ± 0.60	42.28 ± 6.84	26.87 ± 5.13	6.39 ± 3.18	24.46 ± 2.05
	1 mg L^–1^	5597	2	5.33 ± 0.41	24.32 ± 0.98	30.92 ± 4.69	20.09 ± 0.28	24.67 ± 5.64
	10 mg L^–1^	8475	3	6.10 ± 0.57	20.82 ± 8.52	31.16 ± 6.78	20.91 ± 4.08	27.11 ± 4.55
	100 mg L^–1^	5820	25	4.80 ± 0.31	36.68 ± 1.20	33.72 ± 9.58	8.20 ± 3.35	21.40 ± 7.18

a CCN, counting cell numbers; CA, number of chromosomal aberrations, SD, median and standard deviation. Different letters in the same columns for each treatment time show statistically significant differences at *p* ≤ 0.05.

For 1h-LSGO, higher concentrations induced a marked increase in prophase (49.65%) and a concomitant reduction in anaphase percentages (6.95%), indicating a concentration-dependent arrest in early mitosis and disruption of normal cell cycle progression. These effects are consistent with previous reports on GO and related nanomaterials (graphene, GO, and rGO), which have similarly demonstrated that graphene-derived materials (graphene and GO) significantly increase the frequency of micronuclei in primary human lymphocytes at doses of 50 and 100 mg L^–1^.^14,36–39^ In contrast, exposure to 5h-LSGO caused a pronounced decrease in the proportion of cells in prophase (20.82%), accompanied by a relative increase in metaphase (33.72%) and telophase (27.11%) indices, particularly at 10 and 100 mg L^–1^, respectively. This pattern suggests alterations in mitotic spindle assembly and possible incorrect chromosome segregation, primarily in 5h-LSGO at concentrations of 1 and 10 mg L^–1^. At these levels, the percentages of cells in the anaphase (∼20%) and telophase (∼26%) are higher compared to 1h-LSGO, which would facilitate the resolution of cellular reproductive events that translate into root growth.

The MI analysis is presented in Figure 8, where *A. cepa* revealed different cytotoxic responses to 1h-LSGO and 5h-LSGO. For lower concentrations (0.1–1.0 mg L^–1^), both GO variants caused minimal disruption to cell division, with slight variations in the distribution of mitotic phases (Table 4). However, at higher concentrations (100 mg L^–1^), a noticeable decline in MI was observed, particularly in the anaphase and telophase stages, indicating impaired chromosomal segregation and cytokinesis. This suggests that exposure to GO interferes with the normal progression of the cell cycle, especially at high doses (100 mg L^–1^). Similar results were obtained by Yin et al., who demonstrated that the oxidation state, as one of the most important chemical parameters of graphene derivatives, regulates the hemolysis effect on human red blood cells, in hemolytic activity experiments exposed to GO/rGO.^40^

**Figure 8:**
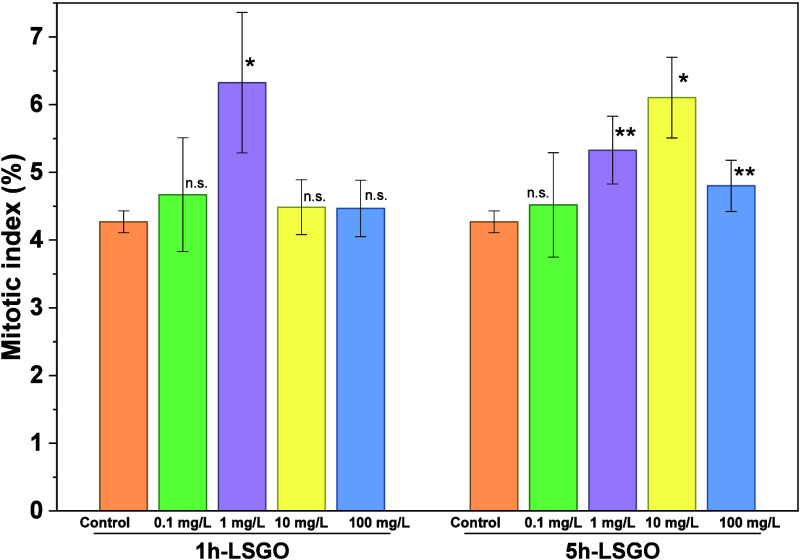
Phase index of (a) 1h-LSGO and (b) 5h-LSGO. Error bars indicate 1 standard deviation. (n.s.): no significantly differences, (*) significant differences at *p* < 0.05, (**) significant differences at *p* < 0.01.

Interestingly, 1h-LSGO, characterized by greater colloidal stability (>−30 mV) with lower sp^2^ content and higher oxygenation, showed moderate cytotoxicity, probably due to greater interaction with the membrane and oxidative stress. In contrast, 5h-LSGO, with greater structural fragmentation, higher sp^2^ bond content, high conductivity (0.485 mS·cm^–1^), and sulfur incorporation for 1h-LSGO (7.2 μM) and 5h-LSGO (7.7 μM), exhibited stronger genotoxic effects at 100 mg L^–1^, which was reflected in reduced mitotic activity and altered phase distribution and consequently lower root growth (Figure 5). When the oxygen percentage is 21.2%, the effect of hemolysis on human red blood cells is approximately 10%.^40^ Meanwhile, in the case of mammalian cells, it has been reported that GOs with a high degree of oxidation (C/O = 2.9) are considered toxic at concentrations of 50 to 100 mg L^–1^.^41^ These findings underscore that the oxygen percentage influences the biological impact of GO, demonstrating that the mitotic index serves as a sensitive biomarker for assessing nanomaterial-induced cytotoxicity in plant systems. Toxicity observed for both materials could be mediated by oxidative stress, as higher concentrations of GO have been associated with elevated production of reactive oxygen species (ROS), including superoxide and hydroxyl radicals, as well as increased lipid peroxidation (more oxygens functional group).^31^ We hypothesize that the higher conductivity in 5h-LSGO promotes oxidative stress via electron transfer, leading to genotoxicity; this is supported by correlations but requires direct ROS measurements (e.g., DCFH-DA fluorescence) in future work.

Figure 9 shows representative micrographs of *A. cepa* root tip cells in different mitotic phases and with typical chromosomal alterations after exposure to 1h-LSGO and 5h-LSGO. Figure 9A–D illustrate normal prophase, metaphase, anaphase, and telophase in control roots, with well-condensed and correctly segregated chromosomes. In contrast, GO-treated roots exhibit several types of chromosomal damage (Figure 9(1)), including anaphase bridges (Figure 9(6)), fragmentation of genetic material (Figure 9(4)), and lagging chromosomes and micronuclei (Figure 9(7)), which are indicative of clastogenic and aneugenic effects. These qualitative observations corroborate the quantitative data (Table 4) obtained from the chromosomal aberration analysis, genotoxicity, and mutagenicity indices presented in the same table, and reinforce the stronger cytogenotoxic potential of 5h-LSGO at 100 mg L^–1^.

**Figure 9:**
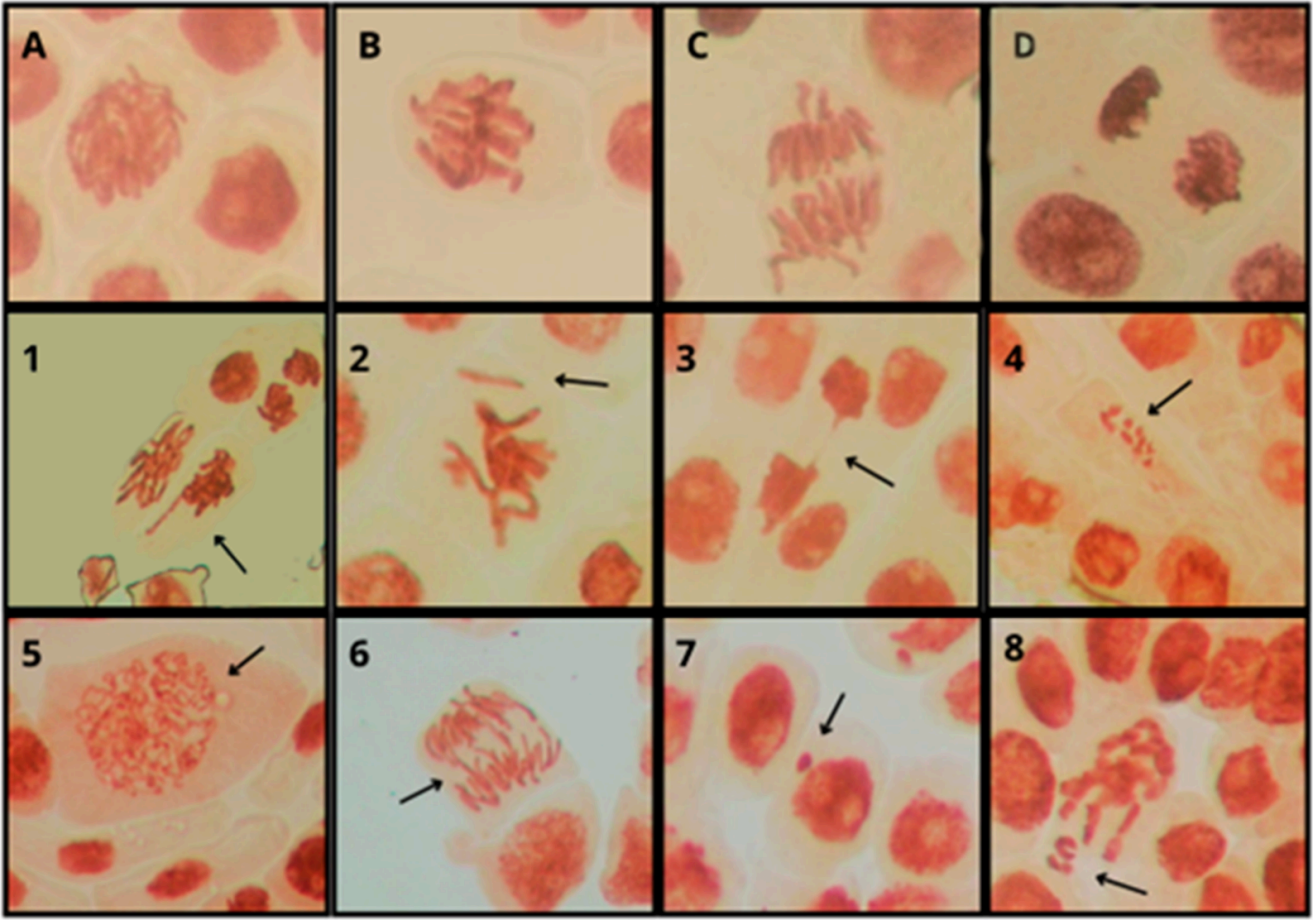
Representative micrographs of *Allium cepa* root meristem cells. (A–D) Normal mitotic phases in control roots (A, prophase; B, metaphase; C, anaphase; D, telophase), showing well-organized and correctly segregated chromosomes: 1—prophase with lagging chromosome; 2—prophase with chromosome loss; 3—telephasic bridge; 4—fragmentation of genetic material; 5—decondensed prophase; 6—anaphasic bridge; 7—micronucleus; 8—metaphase with chromosome loss.

Chromosomal aberration analysis in *A. cepa* root meristems revealed that both 1h-LSGO and 5h-LSGO induced genotoxic effects (Figure 3), particularly at the highest concentration (100 mg L^–1^). While the negative control showed a very low frequency of spontaneous abnormalities, GO-treated roots exhibited a range of structural and numerical alterations, fragmentation of genetic material (Figure 9(4)), lagging chromosomes (Figure 9(1)), anaphase bridges, and micronuclei (Figure 9(7)). The overall frequency of aberrant cells increased with concentration (Table 4), with 5h-LSGO at 100 mg L^–1^ displaying the highest proportion of damaged cells (25 aberrations) compared with the control (3 aberrations), consistent with its stronger impact on the root length relative to control treated (36.56%) and mitotic index (4.80 ± 0.31). A total of 150 aberrations were recorded, represented by lagging chromosomes (16%), chromosomal loss (22%), chromosomal bridges (13%), fragmentations (19%), abnormal chromosomes (18%), and micronuclei (12%) (Table S2, Supporting Information).

Collectively, these cytogenetic parameters confirm that subtle changes in GO surface chemistry and morphology, controlled here by the duration of oxidative interaction with H_2_O_2_, result in distinct genotoxic profiles, with 5h-LSGO exerting a more pronounced cytogenotoxic action on the root meristems of *A. cepa* than 1h-LSGO. This difference is consistent with the markedly higher electrical conductivity of 5h-LSGO (Table 3; 0.485 mS·cm^–1^ at 100 mg L^–1^ versus 0.0249 mS·cm^–1^ for 1h-LSGO), which may facilitate interfacial charge-transfer/redox interactions and thereby exacerbate oxidative imbalance in meristematic tissues.

### 3.6 Are the Tested LSGO Concentrations Environmentally Relevant?

Although graphene and its derivatives have been among the fastest-growing fields of research in nanoscience and technology over the past decade,^42,43^ little is still known about their ecotoxicity at environmentally relevant concentrations. One aspect that should be pointed out is the technical difficulties to obtain reliable GO environmental concentrations: In this line, Goodwin et al. (2018) reviewed the detection and quantification of graphene-family nanomaterials (GFNs) in the environment, but no data on GO concentration in natural waters are included in their study.^44^ Therefore, methods for quantification of GFNs at low concentrations and in complex environmental media and consumer-product-relevant matrices are urgently needed.

A deep bibliographic search was developed to find GO concentrations measured in natural aquatic environments. Unfortunately, the literature review showed that the vast majority of the graphene-related materials (GRM) studies have been carried out under laboratory conditions at concentrations spanning many orders of magnitude (micrograms to milligrams per liter). The lowest 1h-LSGO and 5h-LSGO concentrations tested in our work (0.1 mg L^–1^) are in the range of GO reported by Yang et al. (2020) after spiking GO in water samples, collected from eight different sites in Chengdu city (China).^45^ The authors found GO in the range 0.0011 to 0.2505 mg L^–1^, and Benítez-Martínez et al. (2014) registered GO in the range 0.19–0.39 mg L^–1^ after spiking water samples from the Rabanales River (Spain).^46^ De Marchi et al. (2018) suggest that the predicted environmental concentrations of GRMs are expected to resemble those of carbon nanotubes (CNTs), given their comparable physical and chemical characteristics.^47^ Probabilistic modeling estimated CNT concentrations at approximately 0.17 ng L^–1^ in surface waters, 3.6 ng L^–1^ in wastewater, 0.12 mg kg^–1^ in sewage sludge, and 0.02 ng m^–3^ in the atmosphere. In compartments capable of retaining graphene-based materials, such as soils and sediments, annual accumulations are projected to reach 5.1 ng kg^–1^ and 0.79 μg kg^–1^, respectively.^48^ Analysis developed by Evariste et al. (2020) indicated that GO could induce toxicological effects in top predators as well as in microorganisms at environmentally relevant concentrations.^49^ The highest GO concentration tested by Evariste et al. (2020) is 0.1 mg L^–1^ (equal to the lower concentration tested in this work) and Zhang et al. (2017) exposed zebrafish embryos at GO concentrations from 0.001 to 100 mg L^–1^ (covering all the concentrations tested in this work), to test how the development toxicity changed with the exposure concentrations, and finding the most adverse significant effects in the range 0.1 to 10 mg L^–1^.^50^ Braylé et al. (2022) highlight in their recent review the need to evaluate the impacts of newly introduced GRMs on the market, particularly with respect to their potential effects on microbial communities across diverse environmental matrices.^51^ Nonetheless, additional research is required to comprehensively determine the potential risks and environmental implications associated with LSGO. In this context, this work seeks to make a significant contribution to advancing the understanding of the environmental impacts of GRMs, highlighting their potential risks and emphasizing the urgent need for a thorough evaluation of their effects.

## 5 Conclusions

The physicochemical properties and ecotoxicological behavior of GO were strongly modulated by the oxidative interaction time with hydrogen peroxide (H_2_O_2_). Two materials obtained under identical synthesis conditions, except for H_2_O_2_ interaction time-1h-LSGO and 5h-LSGO, displayed distinct oxidation states and interfacial properties. FTIR deconvolution indicated a higher oxidation degree for 1h-LSGO, with a lower (C=C + H_2_O)/C=O ratio (1.01) and higher oxygen-related bands (ORB, 76.7%) compared with 5h-LSGO (ratio 2.00; ORB 73.6%), consistent with oxygen-group depletion upon prolonged oxidative interaction. In parallel, 5h-LSGO presented a more fragmented morphology (AFM) and a markedly higher electrical conductivity across concentrations, reaching 0.485 mS·cm^–1^ at 100 mg L^–1^ versus 0.0249 mS·cm^–1^ for 1h-LSGO, reflecting its more graphitic/sp^2^-enriched character. Ecotoxicological assays with *L. sativa* and *A. cepa* showed limited effects on seed germination but clear sublethal impacts at the root level. In *L. sativa*, root growth inhibition was more evident for the more oxidized, highly charged 1h-LSGO at the highest concentration. In *A. cepa*, both materials impaired root growth at 100 mg L^–1^, but 5h-LSGO produced stronger inhibition together with a more pronounced decrease in the mitotic index and higher frequencies of chromosomal aberrations.

Collectively, these results demonstrate that relatively small variations in GO surface chemistry, conductivity, and morphology governed here by the H_2_O_2_ interaction time translate into distinct phytotoxic and cytogenotoxic profiles. Notably, the substantially higher conductivity of 5h-LSGO may contribute to its stronger cytogenotoxic. Therefore, precise reporting and control of oxidative treatment conditions are essential for reliable environmental risk assessment and safer application of graphene-based materials in agricultural contexts.

## Supplementary Material



## Notes

Unprocessed AFM height images, raw FTIR spectra of 1h-LSGO and 5h-LSGO, and SEM imaging with lateral size distribution histograms; pseudo-Voigt peak-deconvolution parameters of FTIR spectra; and detailed data on chromosomal aberrations and micronuclei induced in *Allium cepa* root tip cells (PDF)
