# Supplementary material for: Role of Conductivity in the Ecotoxicological Effects of Lyophilized Graphene Oxides on Lactuca sativa and Allium cepa
Source: ACS Omega. 2026 Apr 27;11(18):26797–810. doi: 10.1021/acsomega.5c13552 (PMC13176974; doi:10.1021/acsomega.5c13552)
Supplement: Supplementary file 1 [file ao5c13552_si_001.pdf]

Supplementary material

Role of Conductivity in the Ecotoxicological Effects of Lyophilized Graphene Oxides on *Lactuca sativa* and *Allium cepa*

Wilfredo Rondan Huaman[a*], Maria Rivera[a], Ulises Reno[b,c], Josefina Schmuck[b,c], Luciana Regaldo[b,c], Kauã Habache Farias[a], Jaime Zamora[a], Ana Maria Gagneten[a,b] and Ana Champi[a*].

[a]Laboratorio de Novos Materiais de Carbono: Grafeno.

Universidad Federal do ABC, Santo André, SP 09210-580, Brazil

[b]Laboratorio de Ecotoxicología. Facultad de Humanidades y Ciencias.

Universidad Nacional del Litoral, 3000, Santa Fe, Argentina.

[c]Consejo Nacional de Investigaciones Científicas y Tecnológicas (CONICET), CCT Santa Fe, Santa Fe, Santa Fe. Argentina.

[*]Corresponding authors: *e-mails:* rondan.w@ufabc.edu.br, ana.champi@ufabc.edu.br

Figure S1. Unprocessed AFM and FTIR data for 1h-LSGO and 5h-LSGO. a–b) Representative (unprocessed) AFM height images acquired for 1h-LSGO and 5h-LSGO, respectively (no flattening/leveling or filtering applied). c) Raw (unprocessed) FTIR spectra of 1h-LSGO and 5h-LSGO (no baseline correction or smoothing applied), used for peak deconvolution.

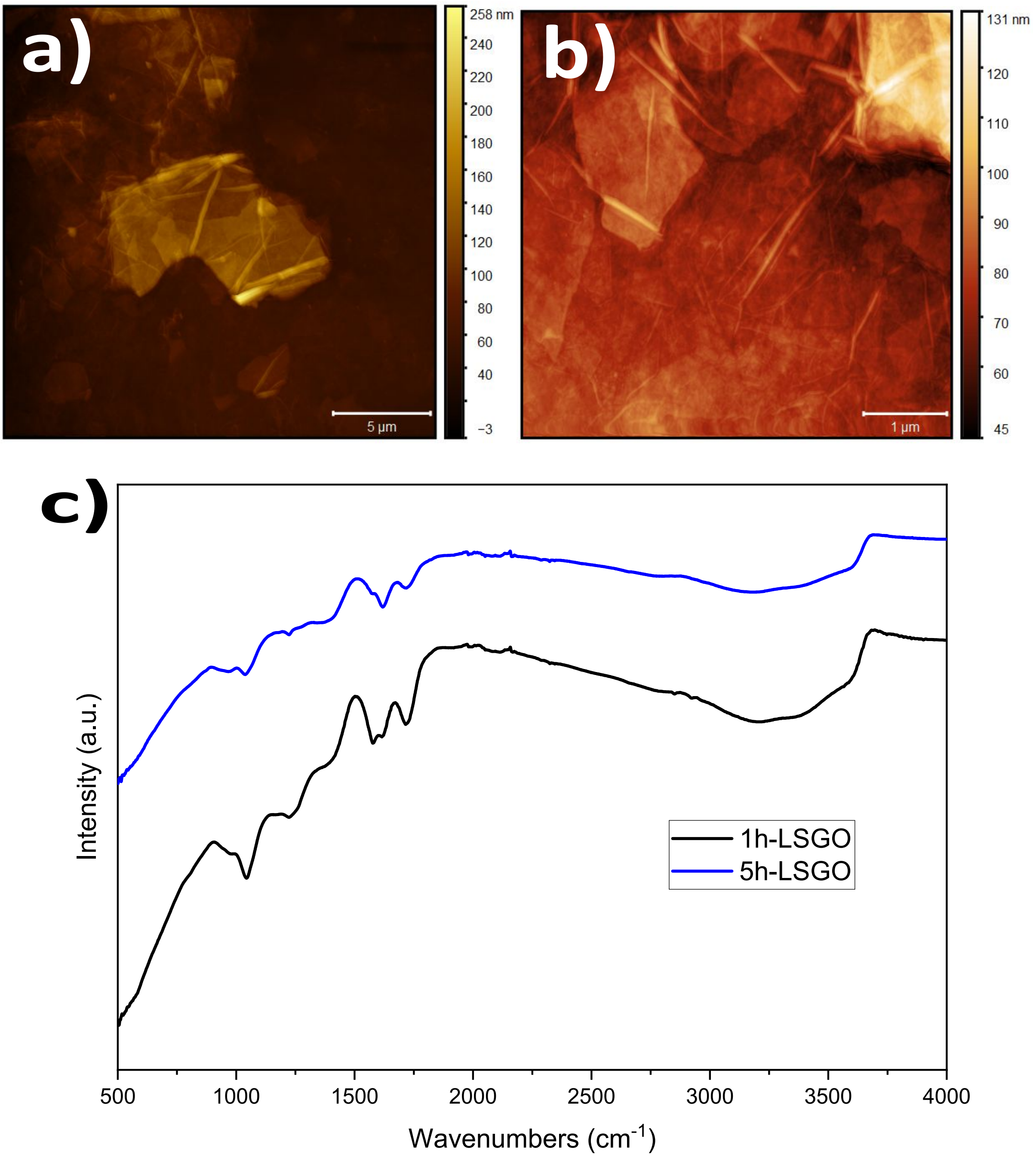

Figure S2. a) Scanning Electron Microscopy (SEM) image of 5h-LSGO graphene oxide and b) lateral size distribution histogram with Gaussian fit, showing a mean size of 0.535 ± 0.192 μm.

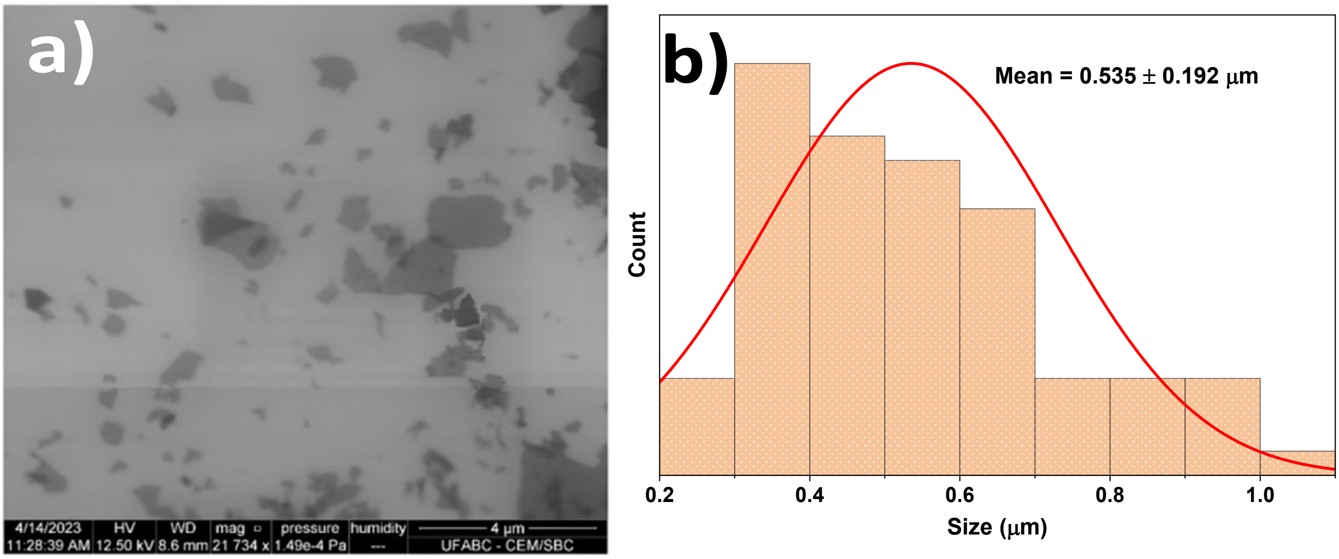

Table S1. Pseudo-Voigt peak-deconvolution parameters for 1h-LSGO and 5h-LSGO FTIR spectra (three-component fit)

Sample	Center (cm^{-1})	FWHM (cm^{-1})	Height (a.u.)	Area (a.u.)	Area (%)
1h-LSGO	1572.9922	46.7123	0.00132162	0.0638671	16.39
1h-LSGO	1621.5745	60.7023	0.00158813	0.131844	33.82
1h-LSGO	1721.6633	78.2039	0.00191918	0.194073	49.79
5h-LSGO	1565.5778	41.7067	0.00064172	0.0223036	6.35
5h-LSGO	1620.5811	65.2931	0.00239316	0.212127	60.36
5h-LSGO	1720.8457	76.7196	0.00136169	0.116978	33.29

Table S2. Chromosomal Aberrations and Micronuclei Induced by 1h-LSGO and 5h-LSGO in Allium cepa Root Tip Cells at Different Concentrations

	1h-LSGO ($mg\ L^{-1}$)					5h-LSGO ($mg\ L^{-1}$)					
**Types of aberrations**	**Control**	**0.1**	**1.0**	**10**	**100**	**Control**	**0.1**	**1.0**	**10**	**100**	**Total**
Lagging Chromosome	0	2	2	2	10	1	1	0	0	6	24
Chromosomal Loss	0	7	1	5	15	0	0	0	0	5	33
Chromosomal Bridges	0	1	3	1	7	1	2	1	0	4	20
Fragmentation	1	6	3	3	11	0	1	0	1	2	28
Abnormal Chromosome	0	5	4	3	7	0	1	0	1	6	27
Micronucleus	3	1	1	4	4	1	0	1	1	2	18
**Total**	4	22	14	18	54	3	5	2	3	25	**150**
**Types of aberrations (%) per LSGO concentration**	**2.67**	**14.67**	**9.33**	**12.00**	**36.00**	**2.00**	**3.33**	**1.33**	**2.00**	**16.67**	**100**

The visual analysis conducted to identify the types of chromosomal aberrations, were as follows.

-Lagging Chromosome: A chromosome that remains stationary at the equatorial plate during migration toward opposite poles of the cell, positioned outside the main mass of migrating chromosomes. The body and edges were clearly defined.

-Chromosome Loss: Represented the initial stage in micronucleus formation. Aneuploidy occurs due to the complete loss of a chromosome, which drifts without direction and remains outside the main chromosomal cluster.

-Chromosomal Bridge: Easily identifiable under microscopy, as it manifests as a literal bridge during cell division. Appearing in the form of a thread or cord, it reflected clear mechanical tension that persisted even after nuclear formation, with the nuclei remaining connected by this filament.

-Fragmentation: A chromosome was broken into fragments that were excluded from the main chromosomal cluster. These fragments drifted without direction.

-Abnormal Chromosome: Chromosomes exhibited atypical morphology, such as being unusually thick, thin, compressed, or excessively decondensed.

-Micronucleus: Small well-defined nuclear body separated from the primary nucleus, typically round or oval in shape, with distinct borders, were detected in some micronucleus, both in 1h-LSGO, and 5h-LSGO samples.
